# 803 Biodegradable Temporizing Matrix as a Dermal Template in the Reconstruction of Pediatric Full-Thickness Foot Injuries

**DOI:** 10.1093/jbcr/irac012.352

**Published:** 2022-03-23

**Authors:** Miriam Renkert, Daniel Svoboda, Lucas M Wessel

**Affiliations:** University Medical Centre Mannheim, Dept. of Pediatric Surgery, Heppenheim, Hessen; University Medical Centre Mannheim, Dept. of Pediatric Surgery, Mannheim, Baden-Wurttemberg; University Medical Centre Mannheim, Dept. of Pediatric Surgery, Mannheim, Baden-Wurttemberg

## Abstract

**Introduction:**

Extensive thermal or traumatic full-thickness injuries to the foot, though rare in children, can lead to severe complications. They are often associated with significant skin loss and underlying tendon or bone exposure. Timely wound closure is essential to minimize the risk of deep infection and to avoid subsequent loss of function.

Tried and tested animal- or human-derived biological dermal templates have always come with a risk of infection and subsequent scar contraction. A biodegradable temporizing matrix (BTM) consisting of a polyurethane scaffold is a synthetic alternative to cover deep defects and promote the development of neodermis.

We report on our first experience with this dermal matrix in the reconstruction of complex foot injuries in children.

**Methods:**

From August 2020 to June 2021, we treated four patients with severe full-thickness injuries of the foot and need for necrectomy with BTM, as the prevalence of exposed bone or tendon prohibited primary wound closure via skin grafting. All cases were caused by contact burns or mechanical trauma. In three of these cases, we utilized a temporary negative pressure wound therapy. Wound closure was achieved by application of full-thickness (3/4) or split-thickness (1/4) skin grafts after delamination of the fully integrated BTM.

**Results:**

Wound monitoring through regular outer dressing changes showed that BTM remained in place and was fully integrated after 3-6 weeks. We saw excellent dermal regeneration in all patients. Swab cultures at first patient contact detected pre-existing wound contamination with Pseudomonas aeruginosa in two children and MRSA in one case. Wound healing before and after skin grafting was unimpaired, though, under topical antimicrobial treatment with polyhexanide. There were no signs of systemic infection.

The take rate of skin grafts was excellent with no graft failure, resulting in pliable, smooth scars. Long-term outcome showed minimal to no scar shrinkage without any functional impairment.

**Conclusions:**

BTM is a good alternative to established dermal templates for complex foot injuries in children. In our experience, even in cases of bacterial wound contamination the synthetic material integrates well and promotes unimpaired tissue regeneration. Contraction and hypertrophic scarring is minimal with very good quality of the developing neodermis.

